# Non-Canonical, Strongly Selective Protein Disulfide Isomerases as Anticancer Therapeutic Targets

**DOI:** 10.3390/biom15081146

**Published:** 2025-08-08

**Authors:** Mary E. Law, Zaafir M. Dulloo, Brian Hardy, Ania Kelegama, Reagan Clark, Mariana Rivas Montbrun, Gabriella Antmann, Srihith Nooka, Ronald K. Castellano, Brian K. Law

**Affiliations:** 1 Department of Pharmacology & Therapeutics, University of Florida, Gainesville, FL 32610, USA; marylaw@ufl.edu (M.E.L.); bhardy1@ufl.edu (B.H.); aniakelegama@ufl.edu (A.K.); reagan.clark@ufl.edu (R.C.); m.rivasmontbrun@ufl.edu (M.R.M.); gabriellaantmann@ufl.edu (G.A.); srihithnooka@ufl.edu (S.N.); 2 Department of Chemistry, University of Florida, Gainesville, FL 32611, USA; zaafirdulloo@chem.ufl.edu (Z.M.D.); castellano@chem.ufl.edu (R.K.C.); 3 UF Health Cancer Center, University of Florida, Gainesville, FL 32610, USA

**Keywords:** protein disulfide isomerases, PDIA1, AGR2, AGR3, ERp44

## Abstract

Protein Disulfide Isomerases (PDIs) are emerging targets in anticancer therapy, with several PDI inhibitors demonstrating anticancer efficacy in preclinical models. Research has largely focused on “canonical” PDIs, such as PDIA1, which contain CXXC active site motifs where C represents Cysteine. Canonical PDIs have well-studied, critical roles in forming, breaking, and exchanging/scrambling disulfide bonds during protein folding. In contrast, non-canonical PDIs, which harbor CXXS active site motifs, remain less well-studied despite their role as sensors or effectors of protein folding quality control during protein trafficking in the secretory pathway. Here, we provide a review of the literature relating to the non-canonical PDIs ERp44, AGR2, and AGR3, which have been identified as strong dependencies in specific cancer subtypes according to the DepMap database. The biological and biochemical functions of ERp44, AGR2, and AGR3 are discussed, highlighting the role of ERp44 in two mechanisms of protein folding quality control, AGR2 as a selective sensor of mucin protein misfolding, and a unique role for AGR3 in cilia. Finally, we discuss recent efforts to develop small molecule inhibitors of ERp44, AGR2, and AGR3 as tool compounds and experimental therapeutics.

## 1. Introduction

Protein synthesis homeostasis, or proteostasis, represents an important therapeutic target in cancer, since tumors are characterized by high rates of protein synthesis driven in part by activation of oncogenes and inactivation of tumor suppressor genes [1,2,3]. This elevated rate of protein synthesis requires high levels of amino acids and frequently exceeds the ability of cancer cells to facilitate native protein folding, resulting in cellular stress responses. Promising anticancer strategies include limiting amino acid levels and exacerbating protein misfolding. Consistent with this approach, depleting asparagine levels using asparaginase is a clinically established treatment for Acute Lymphoblastic Leukemia (ALL) [4,5]. Additional emerging strategies include the use of arginase [6] and inhibitors of amino acid transporters [7,8,9,10,11]. Protein misfolding is implicated in various disease pathologies, including diabetes [12,13,14,15,16] and neurodegenerative disease [17,18,19,20], and is an established characteristic of certain malignancies such as breast cancer [21,22]. With respect to breast cancer, endoplasmic reticulum (ER) stress has been implicated as a therapeutic vulnerability in HER2+ breast cancer [23,24], and more recently in HER2+ breast cancer metastasis [25].

The folding of transmembrane and secretory proteins occurs in the ER. Eukaryotic cells have evolved response mechanisms to protein misfolding in the ER referred to collectively as the Unfolded Protein Response (UPR) or “ER stress” response. The three primary sensors of ER stress are the transmembrane nuclease Inositol Requiring Enzyme 1α (IRE1α) [26,27], the transcription factor ATF6 [28], and the transmembrane kinase Protein Kinase R-like ER Kinase (PERK) [29,30,31]. These sensors initiate signaling pathways that either restore protein homeostasis, or if ER stress is severe and unresolvable, trigger apoptosis, as reviewed previously [32,33,34,35]. ER stress causes PERK-mediated phosphorylation of the translation factor eIF2α, resulting in the suppression of cap-dependent mRNA translation (reviewed in [36]). Similarly, the kinases GCN2, PKR, and HRI are activated and phosphorylate eIF2α in response to amino acid deprivation, double-stranded RNA/viral infection, or heme deprivation, respectively. These pathways collectively form the integrated stress response (ISR), as they converge on eIF2α phosphorylation to regulate protein synthesis [37]. Multiple modes of posttranslational modification promote and maintain native protein folding within the ER, including N-glycosylation, *cis-trans* proline isomerization, and disulfide bonding between cysteine residues. In studies of purified proteins, disulfide bonding and proline isomerization represent rate-limiting steps in protein folding [38,39,40,41,42,43,44], with similar findings observed in studies of protein folding in cultured cells [45,46].

## 2. Protein Disulfide Isomerases as a Druggable Vulnerability in a Subset of Human Cancers

Based on sequence similarity, the human genome encodes 22 members of the protein disulfide isomerase (PDI) family [47] (Table 1). Several recent review articles on PDIs and PDI inhibitors are available [48,49,50,51,52], thus, this review will focus on the subset of PDIs that are inhibited by a series of compounds termed Disulfide bond Disrupting Agents (DDAs). Most PDIs contain either a catalytic CXXC thioredoxin repeat or a non-canonical CXXS thioredoxin repeat, where C, S, and X represent Cysteine, Serine, or any amino acid residue, respectively. The first Cys residue in the thioredoxin repeat is more chemically reactive and initiates the nucleophilic attack on the client protein disulfide bond. The second Cys residue in the canonical CXXC-containing PDIs is termed the “resolving/dissolving” Cys residue and reacts with the first Cys residue of the CXXC motif to cause release of the client protein.

**Figure 1:**
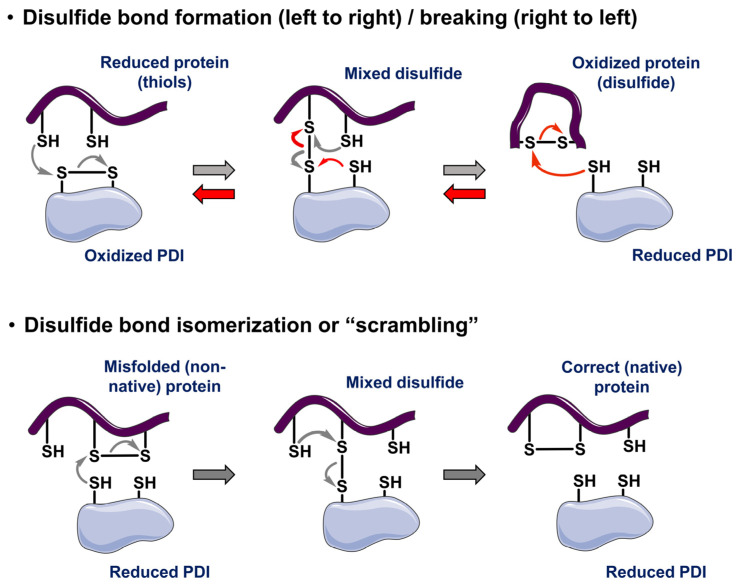
Model for PDIs catalyzing disulfide bond formation (oxidation), cleavage (reduction), or scrambling/exchange with no net change in redox state.

Ser in CXXS-containing PDIs does not react with the first Cys residue causing longer binding times of non-canonical PDIs to their clients. Thus, the CXXC-containing PDIs are considered “foldases”, while the CXXS-containing PDIs are termed “holdases”. In the case of the non-canonical PDIs, another Cys residue in the PDI may attack the Cys residue of the CXXS motif to form an intramolecular PDI disulfide bond and cause client protein release. Alternatively, a Cys residue in the client protein may form a disulfide bond with the client Cys residue bound to the PDI, resulting in an intramolecular disulfide bond in the client and PDI release. A classic study [53] showed that mammalian PDIs containing CXXC or CXXS complemented the loss of yeast PDI, but that in biochemical studies PDI(CXXC) was capable of disulfide bond formation (oxidation), cleavage (reduction), or isomerization/”scrambling”, while CXXS catalyzed disulfide isomerization, but not oxidation or reduction. The observation that the CXXS mutant of human PDI complemented yeast PDI inactivation indicated that, for yeast viability, disulfide isomerization is the essential PDI function. These distinct PDI catalytic functions are highlighted in Figure 1.

Recent studies indicated that the first cysteine (Cys) of the motif initiates nucleophilic attack of client disulfide-bonded Cys residues, while the second Cys is “resolving/dissolving” and mediates substrate release [54]. Consistent with this mechanism, mutation of the second Cys, or in some cases the intervening (XX) residues, generates trapping mutants with prolonged client binding half-lives [55,56]. Interestingly, several human PDIs, including ERp44, AGR2, AGR3, and TMX5, possess CXXS motifs (Table 1), suggesting that these non-canonical PDIs play distinct roles in protein folding compared to PDIs with CXXC active site sequences.

In addition to the thioredoxin repeat, many PDIs contain motifs that anchor them in the ER, preventing their secretion, including transmembrane sequences in the case of TMX1-5, or variations of the C-terminal KDEL sequence. These KDEL and KDEL-like sequences bind to the KDEL receptors 1-3 (KDELR1-3) in the Golgi apparatus and facilitate retrograde trafficking back to the ER [57,58]. The precise functions of KDELR1-3 in the secretory pathway and their degree of redundancy are incompletely understood, making this an area of active investigation.

Given the critical role of protein folding in both cancer cells and in normal tissues, and the mystery of why humans have 22 different PDIs rather than one as in some yeast strains, it is important to determine whether a subset of PDIs is indispensable for cancer cells, but dispensable in normal adult tissues. The Dependency Map (DepMap), developed by the Broad Institute and accessible at depmap.org, employs gene knockout/knockdown technology to classify genes as “common essential” if their inactivation is broadly lethal. In contrast, “strongly selective” genes are those whose inactivation is without effect on most cancer lines and normal cells, but are essential for the survival of a subset of cancer lines. Targeting gene products that are strongly selective for a given cancer may sidestep the systemic toxicities associated with many anticancer medicines. As of 06 June 2025, DepMap data indicate that among the 22 PDIs, only five, ERp44, AGR2, AGR3, PDIA6 and TMX1 are strongly selective based on CRISPR knockout screens. Notably, three of these, ERp44, AGR2, and AGR3 are non-canonical PDIs. Why ERp44, AGR2, or AGR3 are essential for specific cancer cell lines is unclear but may relate to their unique biological and biochemical functions as outlined below.

An important area of future research is to determine the features that control the selectivity of individual PDIs for their spectrum of client proteins. ERp44 binding to and release of its client/partner proteins have been well studied and shown to be controlled by ERp44 cycling between the ER and the Golgi, which differ in their pH, Zn^2+^ concentration, and redox status [59,60,61,62]. How AGR2 client binding is controlled is much less studied than how ERp44 is regulated. While it is clear from AGR2 knockout studies in mice [63], cell culture studies [64,65], and in patients with homozygous AGR2 missense variants [66] that AGR2 is required to sense and respond to misfolding of mucins, little is known regarding the molecular recognition mechanisms. The observation that AGR2 forms mixed disulfide bonds with MUC2 [63] suggests that incomplete disulfide bond formation in mucins plays a role. However, it is possible that other features of misfolded proteins, such as exposure of hydrophobic motifs, also contribute to recognition of misfolded proteins by PDIs.

## 3. Roles of Strongly Selective Disulfide Isomerases in Protein Folding and Cellular Signaling

ERp44 in ER Protein Retention and Protein Folding Quality Control: ERp44 was identified by Sitia and colleagues as a novel ER protein containing a CRFS thioredoxin repeat [67]. Shortly thereafter, ERp44 was shown to retain a subset of clients termed “partner proteins” in the ER by forming a disulfide bond with these clients, thereby preventing their secretion. Examples of ERp44-retained ER partner proteins include ERO1, which generates oxidizing equivalents to drive disulfide bonding in the secretory pathway [68], ER Aminopeptidase 1 (ERAP1), which plays a key role in preparing peptide antigens for presentation to T cells [69], and Peroxiredoxin 4, which reduces hydrogen peroxide to water [70].

ERp44 plays a role in several physiological processes. Through its ability to regulate the secretion or retention of ERAP1, ERp44 may influence both blood pressure and antigen processing for T cell-mediated immunity [71]. ERAP1 controls processing of peptide antigens presented to T cells [72,73,74,75,76] suggesting that the ERp44/ERAP1 cassette may control T cell-mediated immunity in a redox-dependent manner. Additionally, secreted ERAP1 degrades angiotensin II (Ang II), a key regulator of blood pressure [71], indicating that the ERp44/ERAP1 cassette may also modulate blood pressure in response to changes in ER redox status. Depleting ERp44 in db/db mice exacerbates diabetic nephropathy [77], and knockout studies have demonstrated its role in glucose and lipid metabolism [78,79]. Collectively, these findings highlight the involvement of ERp44 in multiple physiological systems.

In addition to its role of retaining partner proteins in the ER, ERp44 has emerged as part of two key checkpoints in protein folding and client trafficking. First, ERp44 was shown to ensure the quality control of disulfide-bonded homo-oligomers, such as immunoglobulin M (IgM) [80,81] and adiponectin [82,83] by recycling misfolded proteins from the Golgi back to the ER via retrograde trafficking. More recently, Tirosh and colleagues discovered selective ER retention (sERr) of specific client receptor proteins whereby mis-disulfide-bonded tyrosine kinases, such as EGFR, MET, and RET, become aggregated in high molecular mass, disulfide-bonded complexes that incorporate ERp44 [84]. sERr is potentiated by PERK inhibitors or ISRIB, which override the integrated stress response (ISR), and permit continued cap-dependent translation in the presence of ER stress.

ERp44 Regulation and Cellular Functions: The localization, conformation, and activity of ERp44, as well as its binding and release of client proteins, are controlled in a complex, integrated manner by the pH [60], zinc [61], and redox gradients that exist between various ER and Golgi sub-compartments. Since these external factors affect the binding/release of ERp44 to/from its clients and partners, these factors have been exploited to some extent in therapeutic or diagnostic experiments. For example, abnormal levels of ERAP1 were attributed to the perturbation of the pH gradient in the ER/Golgi that negatively impacted the interaction between ERp44 and ERAP1.

The role of ERp44 in disulfide-bonding quality control is clear. The cysteine residue C29 in its non-canonical active site (CRFS) is essential for ERp44 to form disulfide bonds with clients, while the cysteine residue, C63, is speculated to participate in the release of clients trapped in a mixed disulfide intermediate state [85,86]. As such, targeting the cysteine residues of ERp44, especially C29, presents an attractive strategy for its inhibition. A related approach is the disruption of ERp44-client complexes. In 2017, Hampe et al. pursued this tactic to improve obesity-related metabolic disorders by modulating the interactions between ERp44 and adiponectin [87]. They synthesized two sets of cell-penetrating peptides (CPPs) modeled after two ERp44 partners, namely, IgM and adiponectin. The synthetic peptides successfully competed with endogenous adiponectin for the ERp44 active site with the goal of releasing adiponectin from ERp44, thus counteracting impaired adiponectin oligomerization and restoring adiponectin secretion. Modulation of the ERp44-adiponectin interaction was more pronounced for the adiponectin-derived CPPs than for the IgM-derived CPPs, a response attributed to their respective binding affinities.

To further investigate the interactions between ERp44 and its clients or partners, the same research group synthesized a brominated variant of the adiponectin-derived CPP, WT36-44-Br [88]. The variant peptide produced an ERp44-peptide mimetic complex by selectively forming an irreversible covalent thioether bond to the C29 residue in the active site of ERp44 as opposed to its free C63 residue. Moreover, the complex was chemically stable under various pH conditions, and was deemed amenable to more extensive structural elucidation by the authors [88]. This approach outlined by Hampe and colleagues could be extended to more recently discovered ERp44 clients and partners such as ATP citrate lyase (ACLY) [89], protein tyrosine phosphatase receptor type-O (PTPR-O) [90], TMX5/TXNDC15 [91], and vascular endothelial growth factor-A (VEGF-A) [92], to study their interactions in greater detail.

Strategies to target ERp44 that extend beyond synthetic peptides include miR-101, a microRNA that targets ERp44 [93]. A negative correlation between miR-101 and ERp44 levels in HTR-8/SVneo trophoblast cells and in human placental tissues with preeclampsia (PE) was consistently observed. Further analysis revealed that miR-101 interacts with the 3′ untranslated region (UTR) of ERp44, and overexpression of miR-101 in trophoblast cells was associated with the downregulation of important ER stress sensors and effectors such as ATF-6, CHOP, caspase-12, and PERK. As a result, the authors posited that miR-101 influences the pathogenesis of PE by modulating trophoblast cell apoptosis through an ERp44-mediated mechanism [93]. Additionally, the small molecule polyphenol, honokiol, was found to alter ERp44 levels [94]. Treatment of oral squamous cell carcinoma (OSCC) cell lines, HN-22 and HSC-4, with honokiol caused degradation of ERp44 in a dose- and time-dependent manner without affecting ERp44 mRNA expression. Honokiol induced apoptosis of tumors in nude mice, as reflected by increased levels of cleaved PARP, with no adverse side effects on body weight. The binding of honokiol to ERp44 was confirmed by pull-down experiments. Moreover, the ERp44-honokiol complex was modeled in silico. Docking studies suggested that honokiol interacts with ERp44 via non-covalent interactions, namely through hydrogen bonds with Glu 365 and Trp 28, and through pi-pi stacking with Arg 372. Collectively, the authors concluded that honokiol can potentially serve as an effective anti-ERp44 agent to treat OSCC [94].

AGR2 and AGR3 are non-canonical disulfide isomerases with intracellular and extracellular functions: Human AGR2 was first discovered as the ortholog of the *Xenopus laevis* cement gland gene Xenopus Anterior Gradient-2 (XAG-2) [95,96]. Both AGR2 and AGR3 contain a single non-canonical (CXXS) thioredoxin repeat and are relatively small compared to other PDIs. AGR2 can undergo two types of dimerization, covalent and noncovalent. Covalent AGR2 dimerization involves a disulfide bond between the Cys residues of two AGR2 molecules [97,98]. Alternatively, AGR2 can form non-covalent dimers [99] in which the Cys residues extend outward from the dimer. The biological significance of these two types of AGR2 dimers is not fully understood; however, covalent AGR2 dimers are incapable of forming disulfide bonds with client proteins. In contrast, non-covalent AGR2 dimers are potentially able to form disulfide bonds with two client proteins simultaneously. The biological importance of AGR2 is perhaps best illustrated by its diverse functions across species. XAG-2 specifies dorsoanterior ectodermal fate [96,100] and contributes to tissue regeneration in amphibians and fish [101,102,103]. In humans, AGR2 deficiency causes inflammatory bowel disease in part because AGR2 is required for production of the mucus proteins that form the colonic barrier that limits intestinal inflammation [66,104]. This observation is recapitulated in AGR2 knockout mice [105]. Additionally, biallelic AGR2 mutations cause a clinical disorder that mimics cystic fibrosis, characterized by decreased production of components of the mucociliary machinery [106].

Several observations suggest that AGR2 plays a role in cancer progression, including its necessity for EGFR presentation at the cell surface [107], its upregulation in Estrogen Receptor-positive breast cancers [108,109,110], and its ability to transform colonic epithelial cells [111]. Some of the documented pro-cancer effects of AGR2 relate to its ER-localized disulfide isomerase activity, which depends on its active site thioredoxin Cys residue and the KTEL C-terminal ER retention motif [112]. AGR2 forms disulfide bonds with EGFR [107], suggesting a role in the folding of EGFR and potentially other HER-family members. Notably, HER1-4 are unique among receptor tyrosine kinases due to their two extracellular Cys-rich repeats per receptor molecule (reviewed in [113]).

Multiple studies show that secreted AGR2 contributes to tumor growth and progression, particularly through its role in angiogenesis [114,115,116] and as a ligand for cell surface receptors, including C4.4A [117] and LYPD3 [118,119]. Moreover, extracellular AGR2 promotes the conversion of non-tumor organoids to tumor organoids, enhancing growth of the organoids approximately ten-fold [120]. Proteins that either promote or inhibit AGR2 dimerization have been identified, indicating a complicated, regulated role for AGR2 in carrying out functions in the ER, cell signaling, and the tumor microenvironment [121].

Recent studies have identified yet another important AGR2 function specific to mucus-producing cells [64,65,122]. AGR2 is one of the top dependencies of multiple colon and pancreatic cancer cell lines, as indicated by the DepMap (depmap.org) (Table 2). Several of these AGR2-dependent cell lines also list components of the Wnt/β-catenin pathway as top dependencies (Table 3). Interestingly, the strongest predictor of cancer cell dependence on AGR2 in the DepMap is the high expression of IRE1β (depmap.org) (Table 4), a paralog of IRE1α that is selectively expressed in mucus-producing cells of the lungs and intestines [64,65]. AGR2 inhibits IRE1β by binding to its luminal domain, thus preventing the dimerization-dependent activation of its nuclease function [64,65]. Ectopic IRE1β expression triggers cell death [31], an effect that is overcome by AGR2 co-expression [64,65]. Although AGR2 disulfide bonding to IRE1β was not detected [64,65], its catalytic Cys 81 is required for binding, suggesting a role for the AGR2 active site in IRE1β binding. Together, these results reveal a Mucin-AGR2-IRE1β axis whereby misfolded mucin sequesters AGR2, de-inhibiting IRE1β, which mediates the response to mucin misfolding. If confirmed, this mechanism would establish AGR2 as a sensor for specific types of protein misfolding.

In contrast to the broader expression pattern of AGR2, AGR3 is expressed in ciliated cells of the epithelium [123], where it regulates ciliary beat frequency. Decreased ciliary beat frequency is associated with impaired mucociliary clearance [123]. Furthermore, AGR3 is overexpressed in several cancers, including colorectal [124], breast [125], prostate [126], and ovarian cancers [127]. While the expression of AGR3 in the serous type of ovarian cancer predicts prolonged patient survival [127], AGR3 expression in colorectal cancer (CRC) predicts poor survival [124], which may be attributed to the ability of AGR3 to promote Wnt/β-catenin signaling and stemness in CRC cells [124].

Therapeutic Strategies Targeting Extracellular AGR2 and AGR2 Production: Targeting PDIs therapeutically presents several challenges. First, the catalytic mechanisms among PDIs are shared with respect to the presence of chemically reactive Cys residues in the context of a thioredoxin-like motif. While these active site Cys residues are obvious targets for therapeutics containing chemically reactive warheads, such agents may also exhibit off-target effects against other proteins with chemically reactive Cys residues. Second, the large number of PDIs present in humans and the possibility of functional redundancy among PDIs make it difficult to know which subset of PDIs is most important to inhibit for anticancer therapy. Given these obstacles, the apparent selectivity of DDAs seems surprising. Of note, the targets of DDAs were initially unknown, thus, optimization of their potency and selectivity was based on their ability to selectively kill cancer cells overexpressing EGFR or HER2 [128,129,130,131]. After identifying the direct DDA targets [132], it became clear that both features of the DDAs themselves and characteristics of PDI subsets dictate DDA selectivity. Discussed previously [133], DDAs can exist in linear or cyclic forms that can interconvert. PDI reaction with a cyclic DDA results in a DDA-PDI disulfide bond and linearizes the DDA resulting in a terminal thiosulfonate group. The DDA thiosulfonate can attack the DDA-PDI disulfide bond, resulting in self-excision of the DDA. This phenomenon may explain how DDAs only bind stably to a small subset of proteins, presumably by stabilizing the linear DDA form and preventing DDA self-excision. Further, since DDAs disulfide bond to the reactive Cys of one of the CXXC repeats, in canonical PDIs, Cys two may form a disulfide bond with Cys one to expel the DDA. However, in non-canonical PDIs that lack Cys two, DDAs may exhibit a much slower off-rate as compared with canonical PDIs. This reasoning explains DDA selectivity for ERp44, AGR2, and AGR3, but does not explain stable DDA binding to PDIA1. Additional variables that include intracellular PDI localization and the pKa and redox potential of their active site Cys residue(s) likely also contribute to the PDI isoform selectivity of DDAs.

As a validated oncogene, AGR2 remains the most thoroughly investigated strongly selective PDI in the literature. However, therapeutic targeting of AGR2 is complicated by the fact that it carries out divergent functions within the ER and extracellular compartments. Various macromolecules, including antibodies, peptides, and microRNAs, have been developed to target AGR2. The mouse monoclonal antibody (mAb) 18A4 is the first AGR2-directed agent capable of selectively binding both recombinant and native AGR2 in cell extracts [134]. 18A4 also inhibits the growth of MCF-7 breast cancer cells in vitro by inhibiting extracellular AGR2. Its therapeutic properties were exploited by Negi et al., who reported that treatment with 18A4 substantially reduced tumor size in A549 and H460 preclinical xenograft mouse models [135]. Additionally, 18A4 suppressed tumor metastasis in the B16-F10 pulmonary melanoma metastasis mouse model, improving survival with no adverse side effects [136]. This study also found that 18A4 activates the tumor suppressor p53, while deactivating ERK1/2-MAPK, a signaling pathway important for cellular proliferation. These findings highlight the involvement of extracellular AGR2 as a regulator of those two pathways and further confirm its role as an oncogene that facilitates cancer cell survival.

To mitigate the risks of immunogenicity associated with the murine mAb 18A4, a humanized variant, mAb 18A4Hu 1, was developed and optimized [136]. This variant preserves its affinity for AGR2 and retains its anti-tumor efficacy against SKOV-3 ovarian cancer xenografts in nude mice. Notably, the study identified two key binding sites, E60-H76 and A86-E153, through which 18A4Hu1 may inhibit AGR2 function [136]. The same group that produced the humanized variant 18A4Hu 1 combined it with an anti-PD1 sequence to produce a bispecific antibody (BsAb) called AGR2xPD1 [137]. The latter exhibited greater anti-tumor activity than its individual components by redirecting lymphocytes to lung cancer cells, reducing the migration of H460 cells, and modulating the interaction between PD1 and its ligand [137]. AGR2xPD1 is currently being investigated in vivo [137].

Anti-AGR2 peptides with therapeutic properties have also been reported. Using an advanced mRNA display technique, Garri et al. screened a vast library of 10^11^ peptides and identified H10 as the strongest AGR2 binding partner with an affinity of 6.4 nM [138]. The binding interface between H10 and AGR2 was carefully studied. Since H10 lacked appreciable affinity toward AGR3, regions with the greatest structural diversity between AGR2 and AGR3 were selected for site-directed mutagenesis. Two amino acids, P41 and E60, that stabilize AGR2 dimerization, and amino acid E96 were revealed as essential participants in H10 binding. These results were supported by in silico docking simulations between H10 and AGR2. Combined data revealed the binding of H10 to AGR2 in its dimeric form. Additionally, attempts to block AGR2 homodimerization, such as decreasing the concentrations of AGR2 to favor its monomeric form or mutating the E60 residue, decreased the binding affinity between H10 and AGR2 [138]. Inhibition of AGR2 homodimer formation is a potential therapeutic strategy pursued by Ullah et al. in a theoretical study in which they successfully disrupted the AGR2 homodimer with five repurposed FDA-approved small molecule drugs that were selected via structure-based screening [139]. The drugs were found to interact with amino acid residues E60-K64 from the AGR2 dimerization domain. Whether the drugs can bind the AGR2 homodimer in vitro and whether this approach holds therapeutic value have yet to be assessed.

Zhang and colleagues designed an anti-AGR2 hexapeptide, NTAIYY, to target pancreatic ductal adenocarcinoma (PDAC) by competitively disrupting the AGR2-RNA polymerase II (RNAPII) complex [140]. NTAIYY was encapsulated in liposomes and tested in vitro and in vivo. NTAIYY consistently produced the desired therapeutic effects on a series of cancer cell lines harboring wild-type p53, including MCF7 and MDA-MB-231 (breast), HCT116 (colon), HT29 (colorectal), A549 and H2087 (lung), and Capan-2 and HPAC (PDAC). Disruption of the AGR2-RNAPII complex in the KC pancreatic ductal adenocarcinoma mouse model led to activation of p53, upregulation of p-H2ax^S139^, and suppression of tumor growth with no adverse side effects on healthy tissues. Additionally, NTAIYY enhanced the sensitivity of wild-type p53-harboring cancers to various therapeutic agents, improving their selectivity and overall cytotoxicity. For comparison, a previously reported hexapeptide, PTTIYY [141], was evaluated alongside NTAIYY, with both exhibiting identical biological activity in vitro and in vivo. Moreover, NTAIYY was mutated at the sixth position to generate NTAIYA, revealing that this tyrosine is essential to effectively bind AGR2 and disrupt the AGR2-RNAPII complex [140]. Any additional interactions between AGR2 and NTAIYY were not investigated.

The third subset of biologics utilized to inhibit AGR2 are microRNAs. In 2018, Pan et al. observed a negative correlation between the expression levels of miR-217 and AGR2 in the tyrosine-kinase inhibitor (TKI)-resistant chronic myeloid leukemia cell line K562DR [142]. This inverse relationship between miR-217 and AGR2 was confirmed as the product of miR-217 binding to the 3′-UTR of AGR2 mRNA, which impacted AGR2 translation and thus protein expression. Consequently, miR-217 overexpression induced therapeutic effects such as reduced tumor burden in the liver and prolonged lifespan of mice transplanted with K562DR through a mechanism involving simultaneous downregulation of AGR2 and sensitization to Dasatinib [142]. Similarly, expression of the miRNA, miR-199a-3p, negatively correlated with AGR2 expression in lung adenocarcinoma tissues in a study performed by Liu et al. [143]. miR-199a-3p was also found to bind directly to the 3′-UTR of the AGR2 gene to repress the proliferation of non-small cell lung carcinoma PC-9 and lung adenocarcinoma Calu-3 cells and to induce cancer cell apoptosis.

Other AGR2 inhibition strategies include: (1) ^125^I seeds, which upregulated p-p38 MAPK and p-p53, induced apoptosis in cholangiocarcinoma cells (CCA), and reduced AGR2 levels in AGR2-overexpressing CCA xenograft tumors [144]; (2) repurposing etravirine, a non-nucleoside reverse transcriptase inhibitor, to downregulate AGR2 via autophagy-mediated lysosomal degradation in both sensitive (A2780) and resistant (A2780ADR) ovarian cancer cells, where it demonstrated therapeutic effects alone and in synergy with paclitaxel [145]; and (3) co-administration of allicin, a sulfhydryl-reactive natural product, and lovastatin, a cholesterol lowering drug, to bypass AGR2-mediated resistance in lovastatin treatment [146].

Inhibition of AGR3: Fewer AGR3 inhibitors are reported compared to AGR2 inhibitors since AGR2 is the more extensively studied PDI of the two. The disparity largely stems from the limited expression pattern of AGR3 in disease states and the resulting uncertainty regarding its pro-oncogenic potential in different cancers. Because AGR2 and AGR3 share structural similarities, owing to a single non-canonical CXXS core thioredoxin motif and their relatively small size, strategies to inhibit AGR2 and AGR3 often overlap. For instance, Gray et al. developed MAGR3-1, a monoclonal antibody with high affinity and selectivity for AGR3 over AGR2, to investigate whether extracellular AGR3 is pro- or anti-oncogenic [147]. The more specific roles of AGR3 in breast cancer and tumorigenesis were further investigated in a study by Obacz and colleagues [148]. The authors found that extracellular AGR3 (eAGR3) promotes breast cancer cell adhesion and migration by participating in the c-Src signaling pathway and by inducing the phosphorylation of tyrosine kinases. Dasatinib, a TKI mentioned previously, was able to control cell migration and to counteract the effects of eAGR3 in T47D breast cancer cells [148]. The relationship between AGR3 and estrogen receptor expression in cancers is intriguing. While AGR3 expression appears to be independent of estrogen receptor status in ovarian cancer [147], a positive correlation was observed in certain breast cancers by Jian et al. [149]. Their study identified AGR3 as an estrogen-responsive gene through Gene Set Enrichment Analysis (GSEA) and demonstrated that AGR3 knockdown reduced cell viability, suggesting a role for AGR3 in promoting the viability of estrogen receptor-positive breast cancer cells. The selective estrogen receptor modulator 4-hydroxytamoxifen also downregulated AGR3 in T47D breast cancer cells [149]. Xu and colleagues examined the role of AGR3 in invasive ductal carcinoma (IDC) and found that AGR3 was predominantly expressed in luminal subtypes, with AGR3 expression associated with various outcomes, including a higher risk of recurrence and metastasis, increased cancer cell invasion and proliferation, and enhanced sensitivity to chemotherapeutic 5-fluoropyrimidines [150]. Although emerging evidence suggests that AGR3 may contribute to the progression of ovarian and breast cancers, its precise role as an oncogene remains unclear.

The development of PDI inhibitors for therapeutic purposes is gaining momentum, with most efforts focused on targeting common essential PDIs. In contrast, strategies to inhibit strongly selective PDIs remain less frequent and typically involve large molecules and biologics targeting extracellular PDIs. As highlighted in this review, there are two cases where small molecules have been employed to target strongly selective PDIs and inhibit their catalytic function [94,146]. Only one of these cases may involve covalent binding of a natural small molecule, allicin, to the active site of AGR2 [146].

## 4. Small Molecule Inhibitors of Strongly Selective and Non-Canonical Disulfide Isomerases

A series of bicyclic thiosulfonate compounds termed Disulfide bond Disrupting Agents (DDAs) were reported to exhibit activity against cancer cells in vitro and breast tumors in vivo [130,131,151,152,153], as well as to induce ER stress and UPR [35]. Recent work identified PDIA1, AGR2, AGR3, and ERp44 as direct cellular targets of DDAs [132]. AGR2 and ERp44 loss-of-function experiments partially mimicked the effects of DDAs on cancer cells, and DDAs were found to disrupt disulfide-mediated AGR2 dimerization, as well as disulfide bonding between ERp44 or PDIA1 and their client proteins. These findings confirm AGR2, ERp44, and PDIA1 as bona fide cellular DDA target proteins. Additionally, DDA treatment causes secretion of ERAP1, a known ERp44 partner protein, from breast cancer cells [132]. Given the role of secreted ERAP1 in lowering blood pressure by mediating Ang II degradation [71] and altering the immunopeptidome [69,76,154] as discussed above, DDAs may hold therapeutic potential not only as anticancer agents but also as antihypertensive or immunomodulatory compounds.

## 5. DDAs: Disulfide Isomerase Inhibitors with a Unique Selectivity Profile

**Figure 2:**
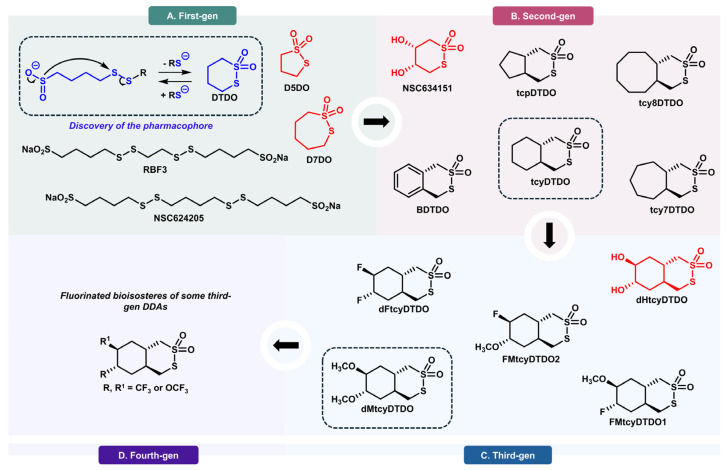
Rational optimization of DDAs across generations. The most potent DDA from each generation is placed in dotted-line boxes, whereas inactive DDAs are shown in red. (**A**) Discovery of the pharmacophore and first cyclic DDA, **DTDO** [128]. (**B**) Bicyclic DDA derivatives and discovery of the most potent second-generation DDA, **tcyDTDO** [130]. (**C**) Decoration of the parent tcyDTDO scaffold and discovery of the most potent third-generation DDA, **dMtcyDTDO** [133]. At this stage of the SAR campaign, a lipophilicity-potency interplay became apparent, supported by the increased potency of **dFtcyDTDO** but lack of activity of **dHtcyDTDO** [133]. (**D**) Incorporation of fluorinated motifs such as CF_3_ and OCF_3_ into the **tcyDTDO** scaffold to create next-generation DDA fluorinated bioisosteres.

To improve the selectivity of DDAs toward their target PDIs, their chemical structures were partially optimized through a structure-activity relationship (SAR) campaign. The pharmacophore, which consists of a disulfide bond separated from a sulfinate anion by a four-carbon linker (highlighted in blue in Figure 2), was first identified in 2015 after screening a series of linear sulfur-containing compounds against various human cancer cell lines [128]. Compounds with the pharmacophore, such as **NSC624205** and **RBF3**, displayed stronger effects toward EGFR+ (MDA-MB-468) and HER2+ (BT-474) breast cancers compared with the pancreatic cancer cell line (BxPC-3). Any structural deviation from this motif resulted in biological inactivity. Field et al., who studied some of these compounds as radioprotective agents [155], pertinently noted that a cyclic thiosulfonate, 1,2-dithiane-1,1-dioxide or **DTDO**, can be released by an intramolecular cyclization reaction involving the pharmacophore [156]. Field’s observation, combined with our recent discovery that certain cyclic DDAs are capable of covalently engaging the active sites of select ER-resident PDIs, strongly supports an intramolecular mechanism of action. Accordingly, the required compound permeability for both the plasma and ER membranes likely results from the uncharged character/lipophilicity of the cyclic form of the pharmacophore, **DTDO**. As expected, both the 5-membered ring (**D5DO**) and 7-membered ring (**D7DO**) showed no biological activity in EGFR- and HER2-overexpressing breast cancer cells since they deviate structurally from the pharmacophore by having a three- and a five-carbon linker, respectively [133]. This difference in biological activity was investigated in silico by Ghilardi et al. and was attributed to a higher chemical reactivity of **D5DO** and **D7DO** toward free thiols and, subsequently, lower selectivity toward PDIs [133]. This chemical reactivity-selectivity dichotomy, whereby **D5DO** and **D7DO** favor their ring-opened forms and off-target binding, is supported by various kinetic and thermodynamic factors detailed in the same work [133]. Hence, **DTDO** was selected as the most potent first-generation DDA.

Initial efforts to generate more potent cyclic DDAs started with the functionalization of **DTDO** at the 4 and 5 positions with hydroxy (**dHDTDO**) and acetoxy (**dAcDTDO**) substituents [129]. Despite the feasibility of these chemical modifications, the resulting DDAs exhibited no biological activity. It is hypothesized that **dAcDTDO** is hydrolyzed by esterases into **dHDTDO,** which is inactive, likely due to a decrease in lipophilicity and membrane permeability. This likely hampers the access of **dHDTDO** to the target PDIs, since PDI inhibitors must traverse both the plasma and ER membranes to reach their protein targets. Subsequently, a series of bicyclic thiosulfonates were prepared [133]. The fused cycloalkane rings were expected to not only increase the lipophilicity of the DDAs but also favor their ring-closing reaction, thus, improving their selectivity by minimizing off-target binding. Theoretical modeling of cyclic thiosulfonate reactivity and DFT simulations indicated that the opened form of bicyclic DDAs is less thermodynamically stable than their closed, monocyclic counterparts, supporting their enhanced self-excision capacity following disulfide-bond formation unless appropriately “trapped” [133]. Overall, bicyclic DDAs exhibit increased potencies and more pronounced biological responses in EGFR+ and HER2+ breast cancer cells [133]. These improvements are attributed to the pre-organization of the reactive end groups, which presumably facilitates ring closure by a pseudo *gem*-dialkyl effect after possible off-target interactions, an effect consistent with our computational studies as well as the independent findings of Bruice and Pandit [157] and Houk and Whitesides [158]. From the library of bicyclic DDAs, the cyclohexane-fused candidate, **tcyDTDO**, was identified as the most potent second-generation DDA and was nominated as the lead compound in the design of third-generation DDAs [133].

At this stage, the results gathered from the SAR campaign rationally prompted the design of lipophilic DDAs. This was achieved through the addition of non-polar substituents to the cyclohexane ring of the **tcyDTDO** scaffold at the more distal 5 and 6 positions to yield **dMtcyDTDO [144]**. This first derivative, bearing two methoxy groups, displayed improved cytotoxicity as indicated by lower IC_50_ and IC_90_ values compared to the parent **tcyDTDO [133]**. Conversely, **dHtcyDTDO,** with two polar hydroxyl groups, was biologically inactive [133]. Guided by the experimental observation that lipophilicity plays a crucial role in DDA potency, we introduced fluorine to the **tcyDTDO** scaffold to generate mono-fluoro (**FMtcyDTDO1** and **FMtcyDTDO2**) and di-fluoro (**dFtcyDTDO**) derivatives [133]. All fluorinated DDA bioisosteres exhibited improved cytotoxicity and more pronounced biological responses in EGFR+ breast cancer cells compared to **tcyDTDO [133]**. Of the three fluorinated DDAs, **dFtcyDTDO** was the most potent [133]. Considering all DDAs prepared in our laboratory, **dMtcyDTDO** stands out as the most potent DDA thus far, likely due to its elevated lipophilicity and the size of its substituents.

Initial observations with first-generation DDA compounds, such as **RBF3**, showed that these agents exhibited toxicity to breast cancer cells in vitro and in animal models without evidence of toxicity [128]. Subsequent work showed that **RBF3** and related DDAs induce ER stress and activate UPR [129]. The second-generation DDA **tcyDTDO** exhibited improved potency for breast cancer cytotoxicity and activation of UPR, efficacy against breast tumors in animal models, and activation of the TRAIL/DR5 extrinsic apoptosis axis [130,131]. The third-generation DDAs **dMtcyDTDO** and **dFtcyDTDO** showed further improvements in potency over **tcyDTDO** with the same observed molecular mechanisms of action and in vivo activity against breast tumors without affecting surrounding normal tissues or altering complete blood cell counts [132,133,151,153]. A second-generation biotinylated DDA identified the PDIs ERp44, PDIA1, and AGR2 as the major DDA targets in cancer cells [132].

DDAs are the first small molecule active site covalent inhibitors of AGR2, AGR3, and ERp44. Mechanistically, we hypothesize that PDI inhibition by the DDAs is initiated by a nucleophilic attack on the thiosulfonate group of DDAs by the thiolate nucleophile from the CXXS motif of “strongly selective” PDIs. This results in a covalently linked DDA-PDI complex containing a ring-opened DDA in a mixed disulfide state. The rate of DDA ring closure and self-excision is, we suppose, reduced after stabilization of the opened DDA by non-covalent interactions with neighboring residues inside the PDI active site pocket, the precise nature of which is unknown but currently under investigation by our group via molecular docking and structure elucidation studies. The reason behind PDI selectivity is somewhat unclear; for instance, why DDAs bind to only one common essential PDI, namely PDIA1, but not to ERp57 or ERp5/PDIA6 which also contain two identical CGHC repeats, is not known. Possible reasons for the observed DDA selectivity may relate to different pKa values and/or redox potentials among PDI active site Cys residues, variables currently under investigation. Nonetheless, the ability of DDAs to act as “tempered electrophiles” that can discriminate between thiolates and can selectively bind certain therapeutically relevant PDIs is strongly supported. A promising future direction is the design and synthesis of trifluoromethyl and/or trifluoromethoxy DDAs since these groups combine the numerous pharmacokinetic benefits of fluorine with molecular sizes that are more comparable to methoxy groups for biologically enhanced fourth-generation DDAs. While biotin-DDA affinity purification studies only identified the PDIs PDIA1, ERp44, and AGR2, future studies with third-generation biotinylated DDA-based probes are needed to ensure that DDAs do not bind PDI-related enzymes, including Selenases such as GPX4.

With respect to future drug discovery, the development of tempered electrophiles holds significant potential to expand the druggable genome [159]. Compounds that utilize both non-covalent interactions and thiol-reactive mechanisms for target recognition have led to innovative approaches in the development of TKIs for cancer therapy, targeting proteins such as JAK3 [160], BTK, [161,162], and EGFR [163,164]. Covalent inhibitors targeting the cancer-specific K-Ras mutant G12C marked a major breakthrough, resulting in the first FDA-approved inhibitors of this previously “undruggable” target (reviewed in [165]). Given the role of cysteine residues in mediating substrate recognition [166], allosteric regulation [167], and catalytic activity [168] of PDIs, tempered or rapidly reversible compounds, such as DDAs, may be well-suited for developing clinical PDI inhibitors. Additionally, DDA-like compounds featuring cyclic thiosulfonates or cyclic disulfides are being developed for other purposes, including thiol-mediated uptake, as demonstrated in the work of Matile and colleagues [169,170,171,172], and bioreductive probes or bifunctional reagents for thiol redox biology, as explored by Oliver Thorn-Seshold and colleagues [173,174].

## 6. Unanswered Questions and Future Directions

**Figure 3:**
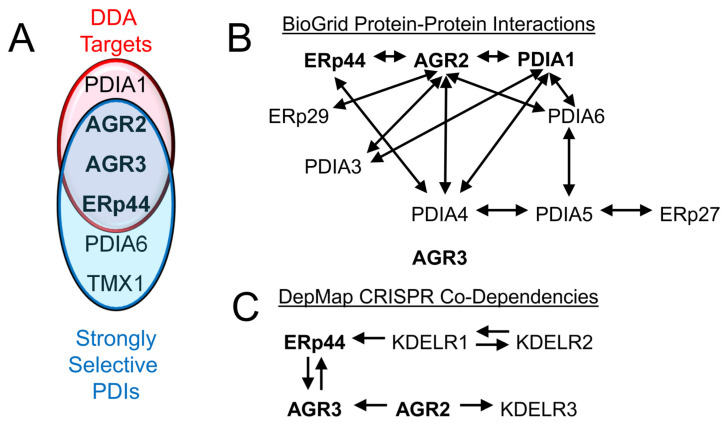
DDA targets and their cancer dependencies, physical complexes with other PDIs, and co-dependencies on each other and KDEL receptors. (**A**) DDA targets identified to date include the PDIs PDIA1, AGR2, AGR3, and ERp44. AGR2, AGR3, and ERp44 are strongly selective PDIs in the Broad Institute’s Map of Cancer Dependencies (DepMap at depmap.org). (**B**) Summary of protein-protein interactions among AGR2, AGR3, ERp44, and other PDIs from BioGrid 4.4 (https://thebiogrid.org/ (accessed on 14 July 2025)). (**C**) Co-dependencies among the strongly selective DDA targets with each other and the KDEL receptors that mediate ER retention. Arrows denote the direction of co-dependency. This information was obtained from the DepMap.

The anticancer activity of DDAs may stem from their ability to inhibit PDIA1, AGR2, AGR3, and ERp44 in parallel (Figure 3A). However, agents that are selective for AGR2, AGR3, or ERp44 would be valuable tools for investigating the specific functions of these proteins and could reduce adverse effects that might be associated with less selective inhibitors. In addition, the mechanisms that determine the selectivity of PDIs for different client proteins are not well understood, and it remains unclear whether the folding of some proteins requires the involvement of multiple PDIs. With respect to ERp44, it will be important to ascertain how its roles in two protein folding checkpoints, sERr and Golgi-ER recycling, are integrated. It is also necessary to investigate whether ERp44 functions only in regulating the trafficking of client and partner proteins or if it also catalyzes disulfide isomerization during protein folding.

Perhaps the least is known regarding why AGR3 is strongly selective against subsets of cancer cell lines. Given its sequence similarity to AGR2, AGR3 may perform similar biochemical functions. It will be important to determine whether double knockout mice lacking both AGR2 and AGR3 display a more severe phenotype than single knockouts, which would suggest some degree of functional redundancy. Since AGR2 is required for proper formation of the mucociliary machinery [64,66,104,122], and AGR3 is required for calcium-mediated regulation of ciliary function [123], double AGR2/AGR3 knockout may exhibit severe lung phenotypes resembling those seen in cystic fibrosis. Interestingly, despite its normally restricted expression, elevated AGR3 levels have been observed in estrogen receptor-positive breast cancers, both within cells and in secreted forms [147,150,175,176], raising the possibility that AGR3 acts as an ER foldase for cell surface receptors, analogous to AGR2. AGR3 may also be secreted and regulate cell surface receptors in a manner like AGR2.

Most cancer therapies, including targeted cancer treatments, involve the use of drug combinations. The approval rate of targeted combination regimens is increasing faster than that of targeted monotherapies [177]. Future studies should focus on identifying inhibitor-based combination regimens that produce strong synergistic effects against cancer cells without elevating toxicity profiles. It is likely that the relevant PDI client proteins will differ across tumor types, and that the mechanisms underlying sensitivity or resistance to PDI inhibitor therapy will vary among different malignancies. There is a need for new PDI inhibitor-based affinity probes designed to monitor how these drugs affect interactions between PDIs and their client proteins. Similarly, PDI inhibitor-based imaging probes are required to track drug distribution within cells, tumors, and throughout the body. Under the appropriate conditions, PDI inhibitors such as DDAs may covalently “trap” or “freeze” binding events.

While most published studies have focused on the roles of PDIs individually, many of these proteins form physical complexes with one another, which are likely to have important biological consequences. For example, the DDA targets PDIA1, AGR2, and ERp44 were found to form non-covalent complexes with each other [132]. This and other observations have led to the concept that PDIs may function as a network to maintain protein folding homeostasis, as proposed by Tirosh and colleagues [84]. Data compiled from the BioGrid website shows that, except for AGR3, all DDA-target PDIs have been observed to associate with at least one other PDI (Figure 3B). AGR2 lies at the opposite end of the spectrum from AGR3, having been detected in associations with six other PDIs. This added layer of complexity, contributed by protein-protein interactions among PDIs, must be considered when analyzing the effects of genetically or pharmacologically ablating the activity of individual PDIs. Another unresolved question is why different PDIs harbor distinct C-terminal ER retention anchors (-KDEL, -RDEL, -KTEL, and -QSEL in the cases of PDIA1, ERp44, AGR2, and AGR3, respectively). The KTEL motif of AGR2 is required for preventing AGR2 secretion and mediating certain AGR2 biological functions, including production of the EGFR ligand amphiregulin and the transcription factor CDX2 [112]. Interestingly, while the ER retention function of KTEL can be mimicked by KDEL or KSEL, these AGR2 mutants lack biological function, indicating a specialized role for the KTEL motif. Another report showed that deletion of the KTEL sequence or mutation of active site residue Cys^81^ to Ser permitted partial AGR2 secretion [178], suggesting that KTEL-mediated ER retention and AGR2 disulfide bonding to ER proteins each play a role in ER retention of AGR2. However, another group observed secretion of wild type AGR2 [120]. These discrepancies might arise from different complements of KDEL receptors 1-3 in various cell lines or from AGR2 disulfide bonds with ER-resident or secreted proteins in different cell lines, or under different conditions. Similar analyses of the role for the active site Cys of AGR3 and its C-terminal QSEL motif in ER retention have not been reported. The DepMap collection of CRISPR co-dependency data in cancer may offer insights into this question, particularly if KDELR1-3 each has distinct sequence preferences for these retention motifs. Notably, ERp44 and AGR3 are co-dependent, and KDELR1 and KDELR2 are co-dependent (Figure 3C). In addition, ERp44 is a KDELR1 co-dependency, while KDELR3 is an AGR2 co-dependency. A more complete understanding of how ERp44, AGR2, and AGR3 facilitate the maintenance of proteostasis will require detailed profiling of their associations and colocalizations with one another and with other PDIs, along with analysis of how this information correlates with the pattern of KDELR1-3 usage by each PDI.

In summary, non-canonical disulfide isomerases appear to mediate distinct biological and biochemical functions. According to current DepMap CRISPR knockout data, three non-canonical PDIs AGR2, AGR3, and ERp44, show selective dependencies, suggesting that they may be promising anticancer therapeutic targets. Although the development of PDI inhibitors as novel therapeutic agents is progressing, efforts remain primarily geared toward the inhibition of common essential PDIs such as PDIA1. A great deal more work is required to fully understand the roles of strongly selective PDIs in normal health and disease and to fully realize their potential as targets for anticancer therapy.

## Figures and Tables

**Table 1:** Human Protein Disulfide Isomerases.

PDIA#	Alternate Names	Catalytic Motif(s)	ER Retention Sequence	Strongly Selective in DepMap (6/16/25)	Confirmed as DDA Targets	Highly Expressed (The Human Protein Atlas; 6/16/25)
1	P4HB	CGHC, CGHC	–KDEL	–	+	Liver, Pancreas
2	PDA2, PDIp	CGHC, CTHC	–KEEL	–	–	Pancreas
3	ERp57	CGHC, CGHC	–QEDL	–	–	Thyroid Gland
4	ERp72	CGHC, CGHC, CGHC	–KEEL	–	–	(Broadly Expressed)
5	PDIR	CSMC, CGHC, CPHC	–KEEL	–	–	Liver
6	ERp5	CGHC, CGHC	–KDEL	+CRISPR	–	(Broadly Expressed)
7	PDILT	–	–KEEL	+RNAi	–	Stomach
8	ERp27	–	–KTEL	+RNAi	–	Pancreas
9	ERp29	–	–KEEL	–	–	(Broadly Expressed)
10	ERp44, TXNDC4	CRFS	–RDEL	+CRISPR +RNAi	+	(Broadly Expressed)
11	TMX1	CPAC	Transmembrane	+CRISPR	–	(Broadly Expressed)
12	TMX2	–	Transmembrane	–	–	(Broadly Expressed)
13	TMX3	CGHC	Transmembrane	–	–	(Broadly Expressed)
14	TMX4	CPSC	Transmembrane	–	–	(Broadly Expressed)
15	TXNDC5, ERp46	CGHC, CGHC, CGHC	–KDEL	–	–	(Broadly Expressed)
16	TXNDC12, AGR1	CGAC	–KTEL	–	–	(Broadly Expressed)
17	AGR2	CPHS	–KTEL	+CRISPR +RNAi	+	Cervix, Intestine, Stomach
18	AGR3	CQYS	–QSEL	+CRISPR	+	Fallopian Tube, Intestine, Lung
19	DnaJC10, DnaJ	CSHC, CPPC, CHPC, CGPC	–KDEL	–	–	Epididymis
PDIB1	Calsequestrin 1	–	–	+RNAi	–	Skeletal Muscle, Tongue
PDIB2	Calsequestrin 2	–	–	+RNAi	–	Heart Muscle
	TMX5, TXNDC15	CRFS	Transmembrane	–	–	(Broadly Expressed)

**Table 2:** Strongly AGR2-Dependent Human Cancer Lines (DepMap; CRISPR). The indicated cell lines listed in DepMap (column 1) have AGR2 as a top-ranked dependency (column 2). The tumor type of origin is listed in column 3 and other top cancer line dependencies in column 4.

AGR2 Selectively Dependent Cancer Line	AGR2 Essentiality Rank (out of 17,347 Genes)	Tumor Type	Other Top 10 Essential Genes (Rank)
C80	1	Colorectal	CDX2 (2)
COLO205	3	Colorectal	FOXP4 (2), FOXA2 (9)
LS513	3	Colorectal	CDX2 (2), CDX1 (5)
SNUC4	5	Colorectal	Axin1 (8)
MAPACHS77	2	Pancreatic	FZD5 (1), WNT7B (3), DVL1 (4), PORCN (6), WLS (7), LRP5 (8)
HSC39	7	Esophagogastric	
2313287	8	Gastric	AXIN2 (9)

**Table 3:** Top AGR2 Co-Dependencies (DepMap; CRISPR). Cancer lines addicted to AGR2 in DepMap exhibit co-dependencies related to transcription and Wnt signaling, the AGR2-related PDI, AGR3, and the ER retention receptor KDELR3.

Rank	Gene
1	SOX9
2	AGR3
3	TTC7A
4	TCF7L2 (Wnt effector)
5	KDELR3

**Table 4:** Top Predictors of AGR2 Dependency (DepMap; CRISPR). List of genes frequently over or under expressed in cancer lines dependent on AGR2 that may serve as predictors of AGR2 addiction.

Rank	Gene	Importance	Corr. Type
1	ERN2 (IRE1β)	22.5%	–
2	MUC3A	2.4%	–
3	LRRC19	1.6%	–
4	SH3BGRL2	1.3%	–
5	TCEA2	1.2%	+
